# Metal–Phenolic Coatings Enable Universal Design of Spherical Nucleic Acids

**DOI:** 10.1002/anie.7693760

**Published:** 2026-06-18

**Authors:** Chaojian Chen, Taokun Luo, Ye Zhang, Xiaowei Liu, Hanwen Zhang, Chad A. Mirkin

**Affiliations:** ^1^ Department of Chemistry Northwestern University Evanston Illinois USA; ^2^ International Institute for Nanotechnology Northwestern University Evanston Illinois USA; ^3^ Department of Materials Science and Engineering Northwestern University Evanston Illinois USA; ^4^ Department of Biomedical Engineering Northwestern University Evanston Illinois USA

**Keywords:** cellular uptake, DNA, metal–phenolic networks, self‐assembly, spherical nucleic acids

## Abstract

Spherical nucleic acids (SNAs), structures consisting of a nanoparticle core chemically modified with a dense shell of nucleic acids, are central to the fields of structural nanomedicine and colloidal crystal engineering with DNA. However, the synthetic methods used to prepare them often require different oligonucleotide immobilization chemistries for each type of core. Here, a general strategy to construct SNAs using metal–phenolic (MP) coatings is introduced. The polymeric shell formed through coordination bonds between polyphenols and metal ions (e.g., Fe^3+^) can be used to modify the surfaces of a wide variety of particles, spanning a diverse range of sizes, shapes, compositions, and surface charges (e.g., gold spheres and cubes, silica, polystyrene, and melamine resin). Importantly, these shells, regardless of nanoparticle core, can be conjugated to thiolated DNA via Michael addition chemistry. MP‐SNAs retain many of the hallmark properties of conventional SNAs, including enhanced cellular uptake, and serve as versatile building blocks for the programmable assembly of a diverse library of nanoparticles and microparticles into larger and more sophisticated superstructures.

## Graphical abstract

A general and straightforward strategy for constructing spherical nucleic acids (SNAs) using metal–phenolic (MP) coatings has been developed. The resulting MP‐SNAs exhibit many of the hallmark SNA properties, including enhanced cellular uptake, and serve as versatile building blocks for the programmable assembly of a diverse library of nanoparticles and microparticles into larger and more sophisticated superstructures.

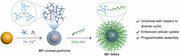

Spherical nucleic acids (SNAs) are a class of nanostructures, typically consisting of a nanoparticle core densely functionalized with a shell of radially oriented nucleic acids. SNAs with cores consisting of metal, metal oxide, silica, quantum dots, proteins, liposomes, lipid nanoparticles, as well as hollow versions of such structures, have been synthesized [1, 2, 3, 4, 5, 6, 7, 8]. Since their introduction in 1996 [9, 10], SNAs have been recognized as a new class of nucleic acids, exhibiting properties that differ markedly from their linear, folded, and circular counterparts with identical sequences [1, 10, 11, 12, 13, 14]. Their three‐dimensional architecture imparts unique characteristics to these conjugate structures, including cooperative binding, resistance to nuclease degradation, and rapid and efficient cellular entry (without the need for transfection agents) [1].

Over the past three decades, these features have enabled SNAs to serve as probes for extra‐ and intracellular detection and agents for gene regulation, gene editing, chemotherapy, vaccination, and immunotherapy [15, 16]. Moreover, SNAs are central to the emerging field of structural nanomedicine [17, 18, 19, 20], where their modular nature allows one to systematically determine how the structural placement of medicinal components influences the potency and safety of therapeutics.

SNAs are also the basis for programmable atom equivalents (PAEs), nanostructured building blocks that underpin the field of colloidal crystal engineering with DNA [14]. PAEs and DNA have been used to prepare thousands of colloidal crystals and superlattices spanning over 90 crystal symmetries [11, 12, 13, 14]. Importantly, some of these structures exhibit properties not observed in nature or by any other known synthetic system, including tunable plasmonic responses and metamaterial behaviors such as negative refractive indices [21, 22, 23].

Despite their broad impact, SNA synthesis often requires core composition‐specific chemistries to attach the DNA strands to the particle surface. Alternative DNA functionalization methods have been explored, including the use of “universal” metallic, silica, or polymeric coatings that simplify DNA attachment [24, 25]. However, some of these methods still require to be tailored based on particle composition, and they are often cumbersome multistep procedures. Therefore, the development of novel, general, core‐independent strategies to construct SNAs could accelerate the use of these materials across nanomedicine, materials chemistry, and other areas of nanoscience and nanotechnology.

Metal–phenolic (MP) polymers [26] have emerged as useful structures for modifying a wide variety of nano‐ and bulk material surfaces [27, 28]. The adhesive nature of phenolic ligands, such as those with gallol and catechol groups that form coordination bonds, hydrogen bonds, and other noncovalent interactions with a wide range of surfaces, makes them remarkably universal in this regard [29]. Consequently, uniform MP coatings can be formed on materials and particles of a wide range of compositions, sizes, and shapes, including ones composed of metals, oxides, and polymers. MP coatings also provide many reactive sites for subsequent functionalization [30]. The phenolic framework can be readily modified with a variety of functional entities, including oligonucleotides, targeting ligands, polymers, imaging agents, and therapeutic molecules, to further expand utility [31, 32]. This generality, chemical modularity, and environmental responsiveness make MP‐coated nanoparticles ideal synthons for preparing SNAs with tunable properties and functions.

Here, MP coatings are harnessed to establish a universal strategy for constructing SNAs that is independent of nanoparticle size, shape, composition, or surface charge. The conformal MP layer deposited on a nanoparticle surface can be subsequently functionalized with nucleic acids via Michael‐addition chemistry. This approach allows for the synthesis of SNAs from metal, inorganic oxide, and polymeric particles using the same protocol. Importantly, MP‐based SNAs have the hallmark characteristics of conventional SNAs [1, 33, 34], including enhanced cellular uptake and sequence‐specific binding that facilitates the programmable assembly of core–satellite superstructures (vide infra) [35, 36].

As a proof‐of‐concept, gold nanoparticles (AuNPs) were selected as the core material (Figure S1). Because AuNPs are a prototypical SNA core, MP–AuNP–SNAs can be directly compared with classical AuNP–SNAs. Briefly, citrate‐stabilized 60‐nm AuNPs were washed twice with Milli‐Q water and redispersed in aqueous solution. To initiate MP coating formation, FeCl_3_ solution (37 mM) was added to the AuNP suspension and vortexed, followed by the addition of tannic acid (TA, 24 mM) and further vortexing (Figure 1A). Upon TA addition, the solution immediately changed from red to blue, indicative of Fe^3+–^TA complexation [37]. Subsequent pH adjustment with 3‐(N‐morpholino)propanesulfonic acid buffer (MOPS, 20 mM, pH 8.0) strengthened the MP coordination interactions, leading to a blue‐to‐purple color change (Figure 1B), a characteristic signature of robust MP network formation [37]. The formation of the MP coating was confirmed by UV–vis spectroscopy (Figure S2), which exhibited a redshift in the plasmon resonance from 535 to 547 nm. Transmission electron microscopy (TEM) showed a uniform coating with an average thickness of ∼12 nm, consistent with typical Fe^3+–^TA films [26]. Energy‐dispersive x‐ray spectroscopy (EDS) mapping further verified the co‐localization of Au and Fe, confirming the presence of the MP layer (Figure S3).

**FIGURE 1:**
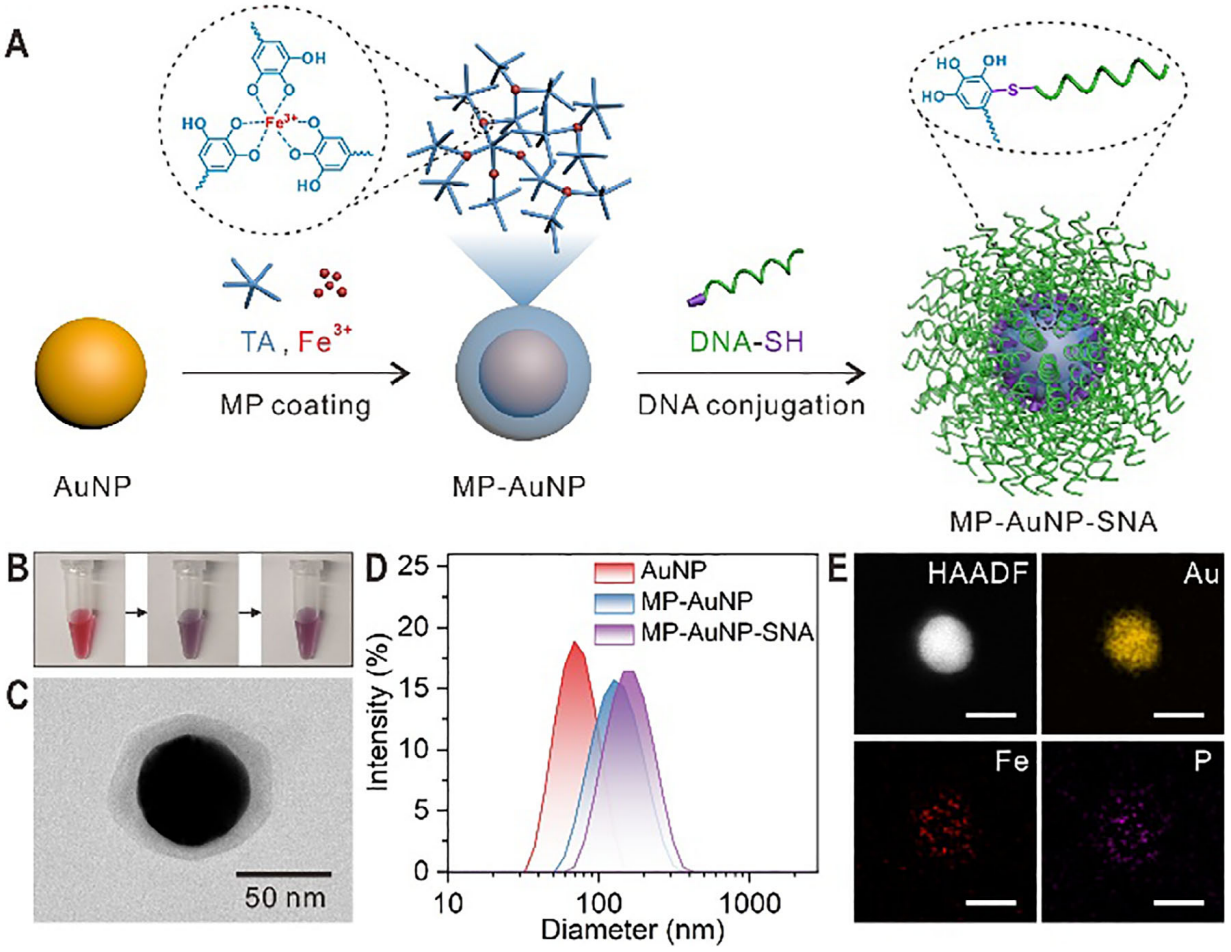
Preparation and characterization of MP–AuNP–SNAs. (A) Schematic of SNA synthesis through nanoparticle coating with MP complexes followed by DNA conjugation via thiol‐based Michael addition chemistry. (B) Photographs showing the sequential color changes from AuNP to MP–AuNP to MP–AuNP–SNA (left to right). (C) TEM image showing the core–shell structure of MP–AuNP–SNA, where the dark core corresponds to the AuNP and the lighter surrounding layer represents the MP coating and DNA modification. (D) DLS size distributions of AuNP, MP–AuNP, and MP–AuNP–SNA. (E) HAADF–STEM image and EDS elemental maps showing the co‐localization of Au, Fe, and P in MP–AuNP–SNA, confirming successful MP coating and DNA functionalization. Scale bars: 50 nm.

Next, hexylthiol‐appended DNA (5SH‐CpG1826, Table S1), freshly treated with dithiothreitol (DTT), was added to the MP‐coated AuNP (MP–AuNP) solution. The ionic strength of the solution was gradually increased to 0.5 M NaCl to facilitate DNA conjugation. Under these conditions, the terminal thiol groups can react with phenolic groups via Michael addition chemistry (Figure 1A) [38, 39]. For comparison, classical AuNP–SNAs lacking MP coatings were also prepared (Figure S4) [9]. Notably, thiol‐based conjugation protocols for Au‐based systems can be directly adapted to MP‐coated nanoparticles, underscoring an advantage of this approach.

DNA functionalization induced an additional redshift of the surface plasmon resonance to 550 nm (Figure S2). The larger redshift relative to that of the classical AuNP–SNAs (plasmon resonance centered at 540 nm) is consistent with a thicker and denser interfacial layer. TEM imaging revealed a distinct core–shell architecture with an average shell thickness of ∼15 nm (Figure 1C), whereas the DNA corona of the AuNP–SNAs was only ∼7 nm (Figure S4). Together, these observations indicate that the intrinsic functionalities of the core materials were largely preserved following the introduction of the MP interlayer and the outer DNA shell. Dynamic light scattering (DLS) measurements showed a stepwise increase in hydrodynamic diameter following MP coating and DNA conjugation (Figure 1D). MP–AuNP–SNA exhibited minimal Fe leaching over 96 h in phosphate‐buffered saline and culture medium (Figure S5), confirming the integrity and stability of the MP coatings under physiological conditions. High‐angle annular dark‐field scanning transmission electron microscopy (HAADF–STEM) coupled with elemental mapping confirmed the co‐localization of Fe and P with Au (Figure 1E), validating the successful conjugation of DNA onto the MP‐coated AuNPs. The surface coverage of MP–AuNP–SNA was quantified by the OliGreen assay to be 460.4 ± 0.8 strands per particle, which is slightly lower than that of AuNP–SNA (565.1 ± 5.4 strands per particle) prepared without the MP coating (Table S2). In addition, particle assemblies generated using complementary DNA showed a melting transition consistent with DNA hybridization, thereby confirming the functionality of the surface‐bound DNA (Figure S6).

To evaluate the generalizability of this strategy, four additional types of particles with varied sizes, shapes, compositions, and surface charges were selected and modified (Figure S7): gold nanocubes (AuNCs, edge length ≈ 80 nm), silica nanoparticles (SiO_2_, 200 nm), polystyrene microparticles (PS, 1 µm), and melamine resin microparticles (MF, 2.1 µm). AuNCs were synthesized according to a reported procedure and stabilized with the cationic surfactant cetylpyridinium chloride [40]. Each particle solution was sequentially treated with FeCl_3_ and TA solutions following the same protocol established for AuNP coating. The formation of MP coatings was evidenced by the characteristic color changes of the particle suspensions (Figure S8) and further confirmed by STEM imaging and elemental mapping (Figures S9 and S10). Notably, the MP coating thickness remained roughly consistent across different particle types, ranging from 15 to 20 nm, as determined by ensemble‐averaged hydrodynamic size distribution from DLS measurements and TEM morphological analysis (Figure 2A). Upon MP deposition, the zeta potential of all the particles shifted to between –28 and –45 mV (Figure 2B), despite the large disparity in their initial surface charges, which ranged from strongly negative (–53.9 mV for PS) to highly positive (+62.9 mV for MF).

**FIGURE 2:**
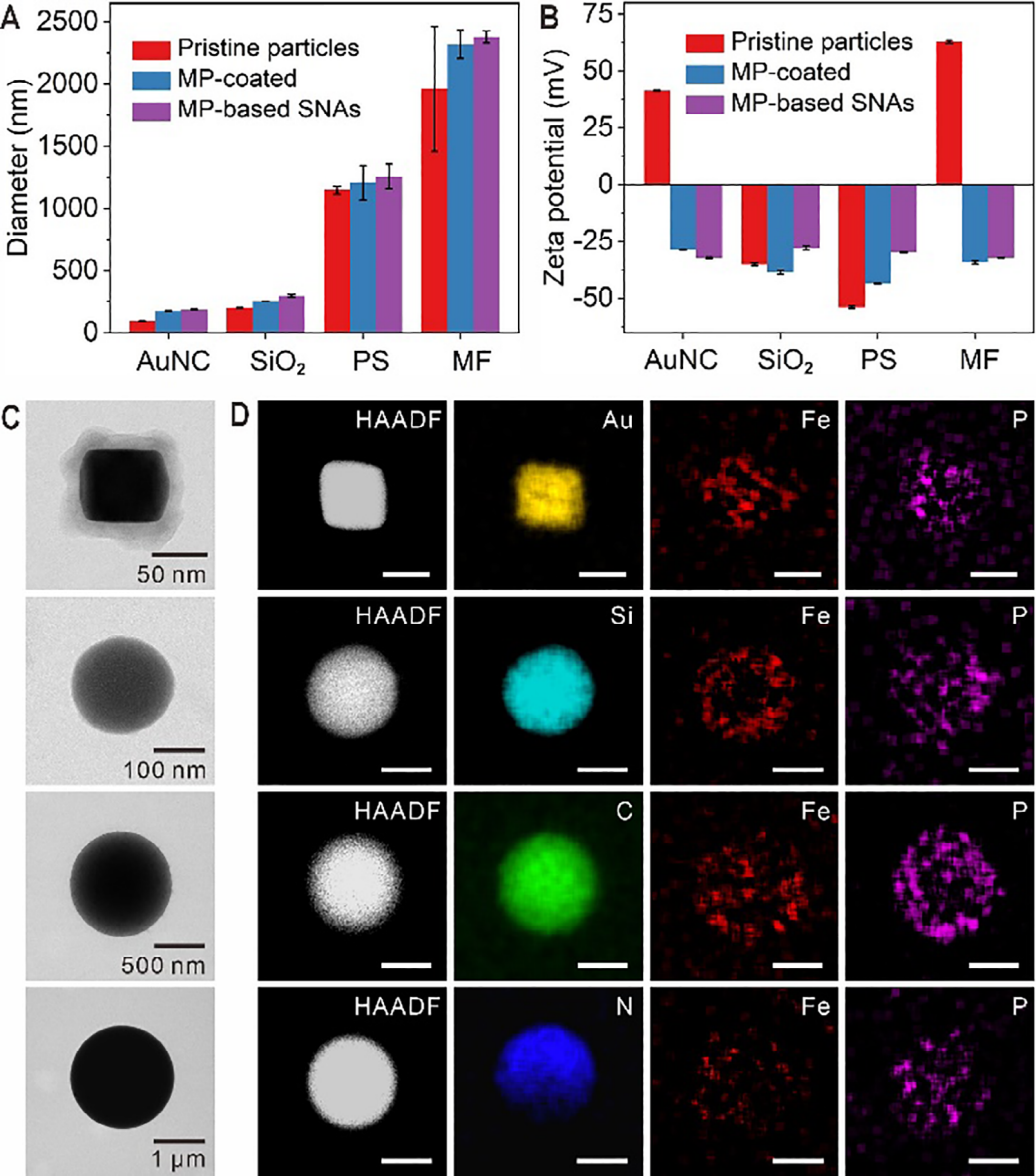
Generalizability of the MP‐based SNA synthesis strategy. (A) Average hydrodynamic diameters of AuNC, SiO_2_, PS, and MF particles before and after modification, measured by DLS. (B) Zeta potential profiles showing surface charge evolution following MP coating and subsequent DNA conjugation. (C) TEM images showing representative particle morphologies (from top to bottom: AuNC, SiO_2_, PS, and MF). (D) HAADF–STEM images and corresponding EDS maps confirming successful DNA functionalization across different particle types. Scale bars in (D) correspond to the respective particles shown in (C).

Subsequently, thiolated DNA was conjugated to the MP‐coated particles following the same procedure established for AuNPs, with the DNA amount adjusted according to the total surface area of each particle type (Table S3). After DNA attachment, the measured hydrodynamic diameters of all samples increased (Figures 2A and S11), and the zeta potential further converged to a narrow range of –28 to –32 mV, indicating that this approach produces SNAs with highly uniform surface characteristics across different cores. TEM imaging (Figure 2C) revealed distinct coating layers on nanoscale AuNC and SiO_2_ particles, while HAADF–STEM and elemental mapping confirmed the co‐localization of characteristic elements (e.g., Fe, P, and elements from the pristine particles), validating successful DNA conjugation onto the MP interfacial layer. Collectively, these results demonstrate that the MP‐based strategy provides a general and effective route for constructing SNAs from nanoparticles of diverse compositions, sizes, and morphologies.

Conventional SNAs are known for their efficient and rapid cellular uptake [1, 34, 41]. To evaluate whether MP‐based SNAs exhibit this property, their cellular internalization was examined using Au, SiO_2,_ and PS particles as representative model systems. The cellular uptake of pristine (bare) AuNPs, MP–AuNPs, and MP–AuNP–SNAs was compared in the DC2.4 mouse dendritic cell line (Figure 3A). After 1 h of incubation, cellular uptake was quantified by inductively coupled plasma mass spectrometry (ICP‐MS). Importantly, MP–AuNP–SNAs exhibited approximately 5.9‐fold higher cellular uptake than pristine AuNPs. In contrast, MP–AuNPs lacking surface DNA showed no significant enhancement relative to unmodified AuNPs, indicating that the increased internalization arises mainly from the DNA corona rather than the MP coating. Notably, AuNP–SNAs without MP layers displayed higher uptake efficiency than MP–AuNP–SNAs (Figure S12), likely due to their higher DNA grafting density (Table S2) [42].

**FIGURE 3:**
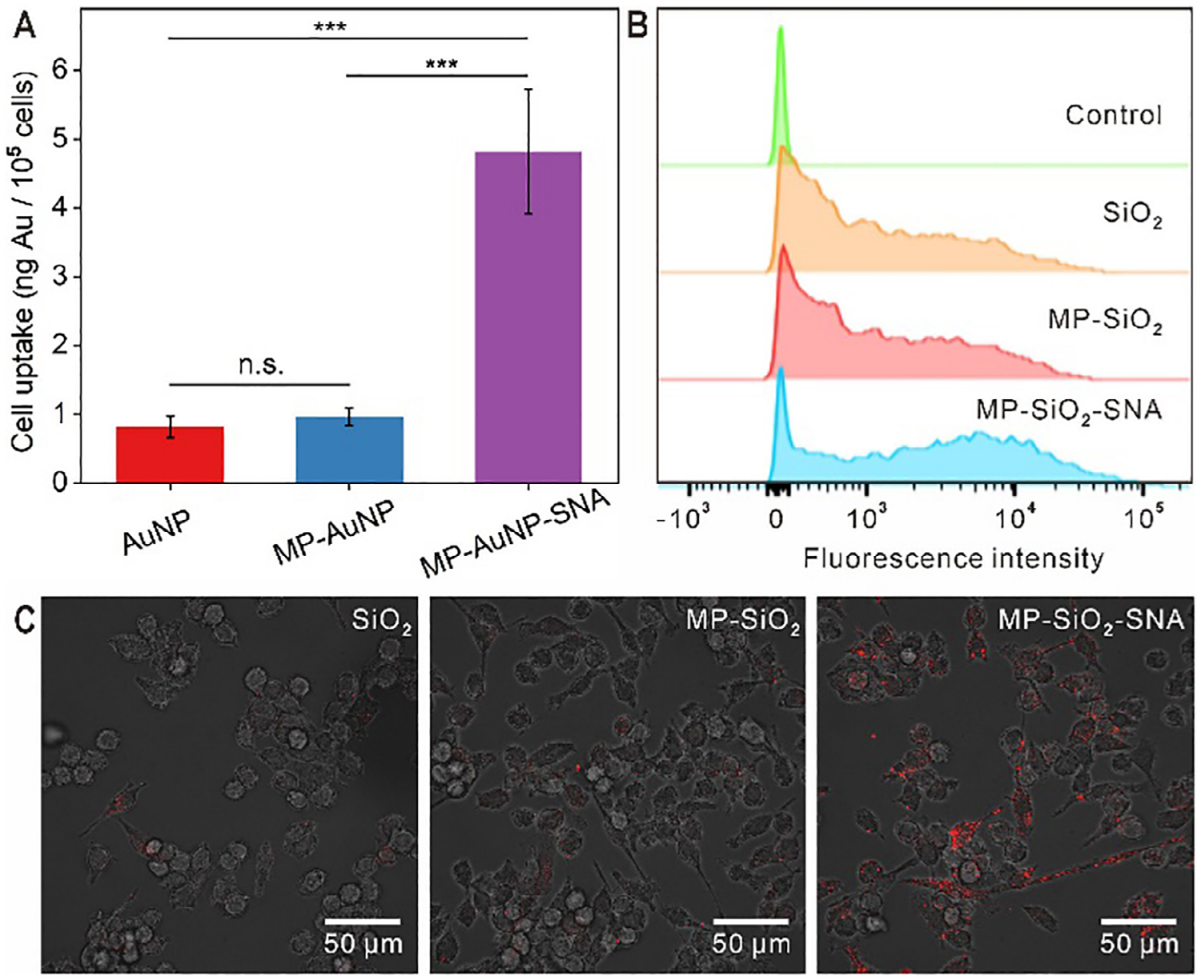
MP‐based SNAs enhance cellular uptake of particles. (A) ICP–MS quantification of cellular uptake for pristine AuNP, MP–AuNP, and MP–AuNP–SNA. Statistical significance was analyzed by one‐way ANOVA with Tukey‐corrected multiple comparisons. ***p < 0.001. (B, C) Uptake of SiO_2_, MP–SiO_2_, and MP–SiO_2–_SNA particles, evaluated by (B) flow cytometry and (C) CSDM.

To further investigate the cellular uptake behavior of other types of MP‐SNAs, fluorescent MP–SNAs were prepared using SiO_2_ and PS particles, enabling quantitative analysis by flow cytometry and direct visualization by confocal spinning disk microscopy (CSDM). The sizes of the fluorescent SiO_2_ and PS particles, as well as the SNA preparation conditions used, were the same as those of their nonfluorescent counterparts. Flow cytometry revealed that MP–SiO_2–_SNAs exhibited markedly higher cellular uptake relative to unmodified or MP‐coated SiO_2_ particles, with the mean fluorescence intensity (MFI) increasing by 3.1‐fold compared to the unmodified control (Figure 3B). Consistent with this observation, CSDM imaging confirmed the efficient cellular uptake of MP–SiO_2–_SNAs (Figure 3C). Quantitative image analysis further showed that the MFI per cell of MP–SiO_2–_SNAs increased by 6.9‐fold relative to unmodified SiO_2_. Similarly, micrometer‐sized MP–PS–SNAs also exhibited significantly enhanced uptake, as supported by both CSDM imaging and flow cytometry (Figure S13). Specifically, the average number of particles per cell increased 1.7‐fold upon SNA conjugation, accompanied by a 2.8‐fold enhancement in flow cytometry signal. These results establish MP functionalization as a general and straightforward route for the preparation of SNAs with diverse compositions and sizes, highlighting the potential of this strategy for applications in bioimaging [4], therapeutics [43, 44], ratiometric sensing [45, 46], and cytosolic nanoparticle delivery [47].

DNA‐functionalized nanoparticles have also been employed as programmable building blocks to construct discrete nanostructures and nanoparticle superlattices, especially in the field of colloidal crystal engineering with DNA [11, 12]. In particular, core–satellite superstructures offer a modular platform for constructing hierarchical nanomaterials with collective optical, electronic, and functional properties, enabling applications in sensing, catalysis, bioimaging, and therapeutics [48, 49, 50, 51, 52]. Therefore, MP–SNAs were explored as programmable synthons for the directed assembly of core–satellite superstructures. MP‐coated MF and PS particles were functionalized with anchor strand A, while 60‐nm AuNPs were modified with anchor strand B (Table S1). Linker strands L_A_ and L_B_ were then hybridized to their respective particles, where L_A_ specifically binds to anchor A and L_B_ to anchor B.

Subsequently, L_A_‐functionalized MF particles were mixed with L_B_‐functionalized AuNPs at defined total surface area ratios (Table S4). The complementary regions of L_A_ and L_B_ mediated sequence‐specific hybridization between the two particle types. Well‐defined core–satellite superstructures with MF cores and multiple AuNP satellites were observed by SEM (Figures 4A and S14). Importantly, varying the particle surface area ratios enabled precise control over the number of AuNP satellites bound to each core. HAADF‐STEM imaging and corresponding EDS elemental mapping (Figures 4B and S15 and S16) confirmed the spatial distribution of AuNPs on the MF particle surface. Similar results were obtained when L_A_‐functionalized PS particles were assembled with L_B_‐functionalized AuNPs (Table S5, Figures S17–S20). Together, these results demonstrate the programmable construction of core–satellite superstructures using MP–SNAs as modular building blocks, establishing a new way of assembling nanoparticles and microparticles into well‐defined architectures for programmable materials design.

**FIGURE 4:**
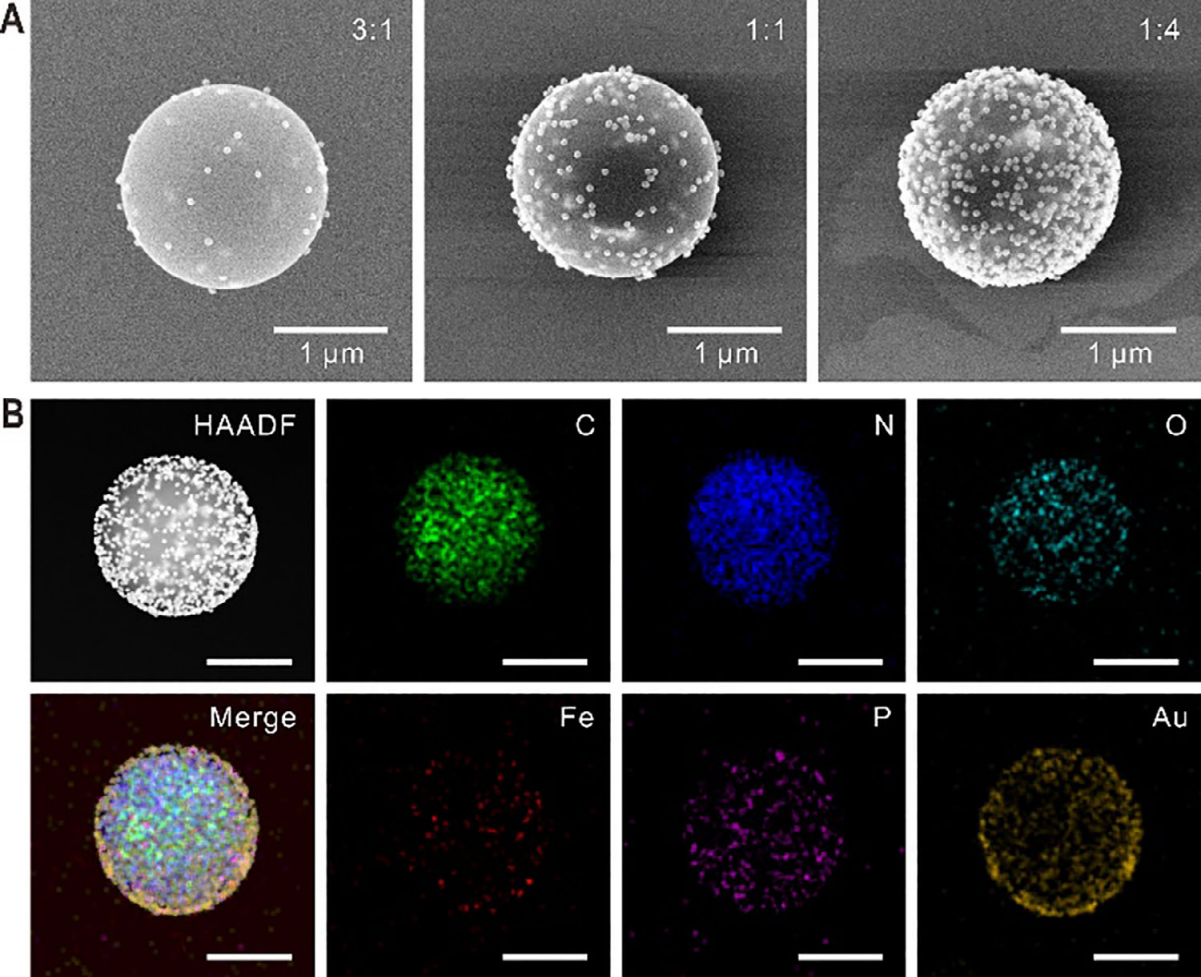
DNA‐programmed assembly. (A) SEM images of MF–AuNP core–satellite superstructures at different surface area ratios, highlighting the effect of particle ratio on assembly density. (B) HAADF–STEM image and EDS maps of a 1:4 MF–AuNP superstructure, showing the spatial arrangement of AuNPs on the MF particle surface. Scale bars: 1 µm.

In summary, we have established a general and facile strategy for constructing SNAs using MP coatings. This universal approach decouples SNA formation from core composition, size, shape, and surface chemistry, thereby enabling the transformation of diverse nanoparticles into functional nucleic acid–grafted architectures. The resulting MP–SNAs preserve the hallmark properties of classical SNAs, including enhanced cellular uptake and programmable assembly capabilities, while offering exceptional modularity and compatibility with existing DNA conjugation chemistries. By uniting the structural versatility of MP networks with the molecular programmability of DNA, this approach provides a promising path for engineering multifunctional nanomaterials. MP–SNAs also open new avenues for the deliberate design of dynamic, programmable nanoscale materials and bioimaging and therapeutic components.

## Author Contributions

**Chaojian Chen**: conceptualization, investigation, writing – original draft, writing – review and editing, visualization, data curation, formal analysis, methodology, validation, project administration. **Taokun Luo**: conceptualization, investigation, writing – review and editing, writing – original draft, methodology, validation, visualization, formal analysis, data curation, project administration. **Ye Zhang**: writing – review and editing, investigation, data curation, formal analysis. **Xiaowei Liu**: writing – review and editing, formal analysis, data curation, investigation. **Hanwen Zhang**: writing – review and editing, data curation, investigation, formal analysis. **Chad A. Mirkin**: conceptualization, funding acquisition, writing – original draft, writing – review and editing, project administration, supervision, resources.

## Conflicts of Interest

The authors declare no conflicts of interest.

## Supporting information



The authors have cited additional references within the Supporting Information [26, 37, 40, 53].**Supporting File**: anie73226‐sup‐0001‐SuppMat.docx.

## Data Availability

The data that support the findings of this study are available from the corresponding author upon reasonable request.
