# Supplementary material for: Metal–Phenolic Coatings Enable Universal Design of Spherical Nucleic Acids
Source: Angew Chem Int Ed Engl. 2026 Jun 18;65(32):e7693760. doi: 10.1002/anie.7693760 (PMC13374119; doi:10.1002/anie.7693760)
Supplement: Supplementary file 1 — The authors have cited additional references within the Supporting Information [26, 37, 40, 53].Supporting File: anie73226‐sup‐0001‐SuppMat.docx. [file ANIE-65-e7693760-s001.docx]

Supplementary Materials for

**Metal–Phenolic Coatings Enable Universal Design of Spherical Nucleic Acids**

*Chaojian Chen,^#,a,b^ Taokun Luo,^#,a,b^ Ye Zhang,^a,b^ Xiaowei Liu,^b,c^ Hanwen Zhang,^b,d^ and Chad A. Mirkin^*,a,b,c,d^*

^a^ Department of Chemistry, Northwestern University, Evanston, Illinois 60208, United States

^b^ International Institute for Nanotechnology, Northwestern University, Evanston, Illinois 60208, United States

^c^ Department of Materials Science and Engineering, Northwestern University, Evanston, Illinois 60208, United States

^d^ Department of Biomedical Engineering, Northwestern University, Evanston, Illinois 60208, United States

^#^ These authors contributed equally to this work.

^*^ Corresponding author: chadnano@northwestern.edu

**The PDF file includes:**

Materials and Methods

Supplementary Tables S1 to S5

Supplementary Figures S1 to S20

References

Materials and Methods

***Materials***

Citrate-stabilized gold nanoparticles (AuNPs, 60 nm, 2.6 × 10^10^ particles/mL) were obtained from Ted Pella. Silica nanoparticles (SiO_2_, 200 nm) were purchased from Sigma-Aldrich and sonicated for 10 min before use to ensure good dispersion. Polystyrene microparticles (PS, 1 µm, 1% solid) were obtained from Fisher Scientific. Melamine resin microparticles (MF, 2.1 µm, 10 wt %), fluorescent silica nanoparticles (200 nm, excitation: 569 nm, emission: 585 nm, 50 mg/mL), and fluorescent polystyrene microparticles (1 µm, excitation: 530 nm, emission: 582 nm, 1% w/v) were purchased from CD Bioparticles. Gold(III) chloride trihydrate (HAuCl_4_∙3H_2_O, 99.9%), cetylpyridinium chloride (CPC, >99%), ascorbic acid (AA, 99%), tannic acid (TA, 98%), iron(III) chloride hexahydrate (FeCl_3_·6H_2_O, 98%), and 3-(N-morpholino)propanesulfonic acid (MOPS, 99.5%) were purchased from Sigma-Aldrich and used as received. Reagents for the oligonucleotide synthesis were purchased from Glen Research.

***Characterization***

UV–vis absorption spectra were recorded over the range of 200–800 nm at room temperature using an Agilent Cary 60 UV–Vis spectrophotometer equipped with a high-precision quartz cuvette (Hellma Analytics; volume: 160 µL; light path: 10 mm; center height: 15 mm). The scanning rate was set to 24,000 nm/min with a data interval of 5 nm. Scanning transmission electron microscopy (STEM) was performed on a Hitachi HD-2300 microscope operated at 200 kV. Dark-field images were acquired using an annular dark-field (ADF) detector. Elemental compositions were analyzed by dual energy-dispersive X-ray spectroscopy (EDS) detectors integrated into the HD-2300 system. Each EDS map was constructed from 30–200 frames with an acquisition time of 10 s per frame. Background subtraction and elemental mapping were processed using Thermo Scientific Pathfinder 2.8 X-ray microanalysis software. Matrix-assisted laser desorption/ionization time-of-flight (MALDI–TOF) mass spectra were collected on a Bruker rapiflex tissue typer using a saturated solution of 2′,6′-dihydroxyacetophenone (DHAP) in methanol as the matrix. Dynamic light scattering (DLS) and zeta potential measurements were carried out on a Malvern Zetasizer Nano. Reported hydrodynamic diameters are based on intensity-weighted size distributions, and all data represent the mean of three measurements. For inductively coupled plasma mass spectrometry (ICP–MS) analysis, samples such as SNAs or cell pellets were first dried under vacuum and then digested with 0.2 mL of a concentrated HNO_3_/HCl mixture (trace metal grade, v/v = 9:1) at room temperature overnight. The digested samples were subsequently diluted with 6.8 mL of deionized water prior to measurement. Elemental concentrations were quantified using an Agilent 7850 ICP–MS after background subtraction. Unless otherwise stated, reported values represent the mean ± standard deviation (SD) from three independent experiments.

***Synthesis of gold nanocubes***

Gold nanocubes (AuNCs, edge length ≈ 80 nm) were synthesized via a seed-mediated growth method following a previously reported protocol.^[1]^ The gold seeds (~25 nm in diameter) were prepared through an iterative reductive growth and oxidative dissolution process. Briefly, the procedure involves: (1) etching of uniformly grown gold nanorods with Au^3+^ ions to yield near-spherical nanoparticles; (2) overgrowth of these near-spherical particles into concave rhombic dodecahedra; and (3) subsequent etching of the concave rhombic dodecahedra into highly uniform spherical nanoparticles. The resulting spherical seeds were dispersed in 100 mM cetylpyridinium chloride (CPC) solution and used for nanocrystal growth. For the growth of AuNCs, 50 μL of the gold seed solution (OD~7) was added to a growth solution containing 10 mL of 100 mM CPC, 1 mL of 100 mM KBr, 200 μL of 10 mM HAuCl_4,_ and 300 μL of 100 mM AA solution. The reaction mixture was gently mixed and allowed to proceed under ambient conditions until the solution color indicated the formation of AuNCs (about 1 hour).

***Metal*−*phenolic coating on particles***

The assembly of metal-phenolic coatings on particles of various sizes, shapes, compositions, and surface charges was carried out following a literature protocol.^[2-3]^ The TA solution (24 mM) was obtained by dissolving 12 mg of TA in 300 µL of deionized water. The FeCl_3_·6H_2_O solution (37 mM) was prepared by dissolving 6.5 mg of FeCl_3_·6H_2_O in 650 µL of deionized water. TA and FeCl_3_·6H_2_O stock solutions were freshly prepared prior to use. A 20 mM MOPS buffer was prepared by dissolving 209 mg of MOPS in 40 mL of deionized water, adjusting the pH to ~8.0 using 1.0 M NaOH, and diluting to a final volume of 50 mL. The MOPS buffer was filtered through a 0.22 µm membrane filter before storage to prolong shelf life.

As a proof-of-concept, 50 µL of a PS particle suspension containing approximately 0.5 mg of particles was transferred into a 1.7 mL microcentrifuge tube, diluted with 500 µL of deionized water, and dispersed by vortexing and sonication for 1 min. The particles were pelleted by centrifugation (2000 rcf, 2 min), and 500 µL of the supernatant was carefully removed. This washing step was repeated once, if necessary, to ensure complete removal of residual surfactants or stabilizers. The washed particles were resuspended in 440 µL of deionized water and sonicated for 1 min to achieve uniform dispersion.

To initiate metal−phenolic coating formation, 5 µL of the FeCl_3_·6H_2_O solution was first added to the particle suspension and vortexed for 10 s, followed by the addition of 5 µL of the TA solution with another 10 s of vortexing. This resulted in the formation of 500 μL of a blue suspension, indicating the formation of a metal−phenolic complex. Subsequently, 500 µL of MOPS buffer (20 mM, pH 8.0) was added to adjust the reaction pH, followed by shaking for 5 min. A slight color change from blue to violet was observed, confirming successful coordination-driven film assembly.

The MP-coated particles were washed three times by centrifugation with Milli-Q water to remove excess precursors. After the final wash, the MP-coated particles were stored in deionized water or in a desired buffer solution until further use. Coating of other types of particles followed the same general procedure with appropriate adjustments to particle amount and centrifugation conditions.

***DNA design, synthesis, and purification***

Oligonucleotide sequences were designed prior to synthesis (Table S1). DNA synthesis was performed on a Mermade 12 (MM12) DNA synthesizer. The oligonucleotides were cleaved from controlled pore glass (CPG) supports using a 1:1 volume mixture of 30% ammonium hydroxide and 40% aqueous methylamine (incubation at 55 °C for 30 min). The solvents were then evaporated, and the crude oligonucleotides were purified using reverse-phase high-performance liquid chromatography (RP-HPLC) on a Varian Microsorb C18 column (10 μm, 300 × 10 mm). The dimethoxytrityl (DMT) protecting groups were removed by treatment with acetic acid and ethyl acetate solutions. The purified oligonucleotides were characterized by MALDI-TOF mass spectrometry to verify their molecular masses. Sp18 represents the hexaethyleneglycol spacer synthesized from 18-O-dimethoxytritylhexaethyleneglycol,1-[(2-cyanoethyl)-(N,N-diisopropyl)]-phosphoramidite. SH represents the hexylthiol group synthesized from 1-O-dimethoxytrityl-hexyl-disulfide,1'-[(2-cyanoethyl)-(N,N-diisopropyl)]-phosphoramidite.

| **DNA Type** | **Sequences** |
| --- | --- |
| 5SH-CpG1826 | SH (Sp18)2 TCC ATG ACG TTC CTG ACG TT |
| Anchor strand A | TCA ACT ATT CCT ACC TAC (Sp18)2 SH |
| Anchor strand B | TCC ACT CAT ACT CAG CAA (Sp18)2 SH |
| Linker A (L_A_) | GTA GGT AGG AAT AGT TGA Sp18 TAGCTA TCTCT |
| Linker B (L_B_) | TTG CTG AGT ATG AGT GGA Sp18 AGAGA TAGCTA |

**Table S1.** DNA sequences used for the functionalization of various particles.

***Fe release profiles of MP-AuNP-SNA***

To evaluate the stability of the MP coatings, Fe release from MP-AuNP-SNA was examined in 1×PBS and Dulbecco’s modified Eagle medium (DMEM). Briefly, 20 μL of freshly prepared MP-AuNP-SNA was mixed with 100 μL of buffer and incubated at 37 °C under stirring. At predetermined time intervals (24, 48, 72, and 96 h), the samples were centrifuged, and the supernatant (100 μL) was collected for ICP-MS analysis to quantify the Fe concentration.

***Preparation of AuNP-SNA***

AuNPs were functionalized with 5SH-CpG1826 (Table S1) following a previously reported procedure.^[4]^ Briefly, hexylthiolated oligonucleotides were treated with 100 mM dithiothreitol (DTT) in 170 mM sodium phosphate buffer (pH 8.0) for 1 h. Residual DTT was removed using NAP-10 size-exclusion columns (GE Healthcare), and the purified DNA was immediately added to the AuNP suspension (5 nmol of DNA per mL of nanoparticle solution).

Phosphate buffer (0.1 M, pH 7.4) and 0.1 wt% sodium dodecyl sulfate (SDS) were then added to each particle solution, which was briefly sonicated and placed on a shaker at 1,200 rcf overnight. Subsequently, 2 M NaCl containing 10 mM phosphate buffer (PB) was added stepwise every 30 min to gradually increase the ionic strength to 0.05, 0.1, 0.2, 0.3, 0.4, and 0.5 M NaCl, with brief sonication (30 s) after each addition. After the final addition of NaCl, the suspension was shaken again at 1,200 rpm overnight to achieve dense DNA loading.

The resulting DNA-functionalized AuNPs were purified by three sequential centrifugation cycles, with the supernatant replaced each time by 0.5 M NaCl and 0.01 M phosphate buffer (pH 8.0). After the final wash, the AuNP–SNAs were redispersed in phosphate-buffered saline (PBS; 0.5 M NaCl, 10 mM sodium phosphate buffer) for storage and further use.

***DNA conjugation to MP-coated particles***

The DNA functionalization of MP-coated nanoparticles was performed using a procedure similar to that used for AuNP–SNAs, with the amount of DNA adjusted to the particle surface area (Table S3). For instance, 50 µL of pristine PS particles (0.5 mg) required 50 nmol of DTT-cleaved DNA for incubation with the MP-coated PS suspensions.

After DNA addition, the particle suspensions were gradually brought to 0.5 M NaCl through stepwise salt addition, followed by shaking overnight at 700 rpm to facilitate DNA adsorption and organization on the surface. The resulting MP-based SNAs were washed three times with deionized water and finally redispersed in PBS (0.5 M NaCl, 10 mM sodium phosphate buffer) for storage and subsequent use. Notably, SDS was omitted in this procedure, as the MP coating improved the colloidal stability of the particles during DNA conjugation.

Single-stranded DNA (ssDNA) loading on 60 nm gold nanoparticle spherical nucleic acids (AuNP–SNAs) and metal–phenolic SNAs (MP–AuNP–SNAs) was quantified using the Quant-iT^TM^ OliGreen^TM^ ssDNA Assay Kit (Thermo Fisher Scientific). Gold nanoparticle cores were first digested with potassium cyanide. Briefly, 0.5 nM AuNP–SNAs or MP–AuNP–SNAs were diluted to a final volume of 50 µL and mixed with 50 µL of 40 mM potassium cyanide (KCN). Samples were incubated at 55 °C for 20 min until complete dissolution of the gold cores, as indicated by sample clarification. Following digestion, 50 µL of the resulting solution was diluted with 50 µL of deionized water and combined with 100 µL of 0.5% Quant-iT^TM^ OliGreen^TM^ reagent (Invitrogen) prepared in 1× TE buffer. Fluorescence was measured in a 96-well plate (λ_ex = 480 nm, λ_em = 520 nm) using a BioTek Cytation 5 imaging reader and quantified against a standard curve generated from the corresponding ssDNA. The ssDNA concentration was then normalized to gold nanoparticle concentration to determine DNA loading per particle.

| **Groups** | **DNA loading density (strand#/particle)** |
| --- | --- |
| AuNP–SNA | 565.1±5.4 |
| MP–AuNP–SNA | 460.4±0.8 |

**Table S2.** DNA surface grafting density of AuNP–SNA and MP–AuNP–SNA quantified by the OliGreen Assay (N=3).

| **Particle** | **Size** | **Weight (mg)** | **Particle surface area (nm^2^)** | **DNA (nmol)** |
| --- | --- | --- | --- | --- |
| AuNP | 60 nm (diameter) | 0.284 | 1.471×10^15^ | 25 |
| AuNC | 80 nm (edge length) | 0.4 | 1.553×10^15^ | 27 |
| SiO_2_ | 200 nm (diameter) | 0.25 | 2.828×10^15^ | 50 |
| PS | 1 μm (diameter) | 0.5 | 2.857×10^15^ | 50 |
| MF | 2.1 μm (diameter) | 1 | 1.938×10^15^ | 35 |

**Table S3.** Conditions used for the DNA conjugation of MP-coated particles.

***Cellular uptake of AuNP-based SNAs***

DC2.4 cells were maintained in complete RPMI-1640 supplemented with 10% FBS and 1% penicillin-streptomycin at 37 °C and 5% CO_2_. Cells were seeded at 1.0 × 10^5^ cells per well in 12-well plates. After overnight culture, different nanoparticles, including AuNP, MP-AuNP, MP-AuNP-SNA, and AuNP-SNA, were added to cells at an equivalent mass concentration of 2 μg/mL. Following 1-h incubation, cells were washed 3 times with PBS, detached by trypsinization, and collected by centrifugation (300 rcf, 5 min). Supernatants were removed carefully, and pellets were dried under vacuum and digested by acid for ICP-MS analysis. Elemental concentrations were converted to Au mass per well and normalized to cell number (counted by Vi-CELL BLU) to report ng Au per 10^5^ cells.

***Cellular uptake of SiO_2_- and PS-based SNAs***

For fluorescence-based studies, 200 nm fluorescent SiO_2_ (Ex/Em 569/585 nm) and 1 µm fluorescent PS (Ex/Em 530/582 nm) particles were used. For flow cytometry, DC2.4 cells were seeded at 1 × 10^5^ cells per well in 12-well plates. Cellular uptake of fluorescent SiO_2_ and PS particles (pristine, MP-coated, and MP-based SNAs) was quantified on a BD Symphony A3 flow cytometer. Cells were incubated at an equivalent particle concentration of 2 μg/mL for 1 h as described above, washed 3 times with PBS, detached, passed through 40 µm filters to obtain single-cell suspensions in PBS + 0.5% bovine serum albumin (BSA), and measured by flow cytometry. For confocal imaging, DC2.4 cells were seeded at 1 × 10^5^ cells per well in glass-bottom dishes (35 mm, MatTek) and treated with the particles in the same way as flow cytometry. After a 1-h incubation, the DC2.4 cells were washed three times with PBS and fixed with 4% paraformaldehyde for 15 min. Images were acquired on a Nikon Sora spinning-disk confocal microscope.

***DNA-mediated particle assembly***

For the DNA-mediated particle assembly, MP-coated PS or MF particles were functionalized with Anchor strand A, while 60 nm AuNPs were functionalized with Anchor strand B. After immobilization of the hexylthiolated anchor strands, linker strands L_A_ and L_B_ (Table S1) were hybridized to their respective particles, where L_A_ specifically binds to Anchor A, and L_B_ to Anchor B. The hybridization was carried out at 55 °C under shaking at 1000 rpm for 30 min, followed by gradual cooling to room temperature. The resulting DNA-conjugated particles were washed by centrifugation and redispersed before use.

For assembly, the core particles (PS or MF) and AuNP suspensions were first adjusted to the desired concentrations and then mixed at predetermined surface-area ratios (Tables S4 and S5). The mixture was then heated to 70 °C for 30 min under shaking at 800 rpm, followed by slow cooling (0.1 °C/min) to room temperature to promote maximal hybridization between complementary strands. The complementary regions of L_A_ and L_B_ enabled sequence-specific hybridization between the two particle types, driving the formation of core–satellite superstructures. After assembly, the hybrid structures were gently purified by centrifugation and collected for STEM characterization.

Thermal UV–vis experiments were performed using a Cary 5000 UV–vis–NIR spectrometer to determine the melting temperatures of MP-SNA assemblies. The assembly solution, with a total volume of 1 mL, was prepared in quartz cuvettes under magnetic stirring. The solution contained MP-functionalized 60 nm gold nanoparticles, corresponding linker DNA strands, and 0.1 M NaCl. The sample was heated to above 70 °C and then cooled to 25 °C at a rate of 0.2 °C/min. During the slow cooling process, the absorbance at the localized surface plasmon resonance peak of the 60 nm gold nanoparticles was monitored at 545 nm.

| **Surface area ratio (MF:Au)** | **MP-MF-SNA (μL)** | **AuNP-SNA (μL)** |
| --- | --- | --- |
| 3:1 | 100 | 100 |
| 1:1 | 33 | 100 |
| 1:4 | 8 | 100 |

**Table S4**. Conditions for the DNA-mediated assembly of MF and Au particles. The concentration of MP-MF-SNA was 3.650 mg/mL (surface area: 7.062×10^15^ nm^2^/mL). The concentration of AuNP-SNA was 0.454 mg/mL (surface area: 2.354×10^15^ nm^2^/mL).

| **Surface area ratio (PS:Au)** | **MP-PS-SNA (μL)** | **AuNP-SNA (μL)** |
| --- | --- | --- |
| 5:1 | 100 | 100 |
| 2:1 | 40 | 100 |
| 1:5 | 4 | 100 |

**Table S5**. Conditions for the DNA-mediated assembly of PS and Au particles. The concentration of MP-PS-SNA was 2.058 mg/mL (surface area: 1.177×10^16^ nm^2^/mL). The concentration of AuNP-SNA was 0.454 mg/mL (surface area: 2.354×10^15^ nm^2^/mL).

Supplementary Figures


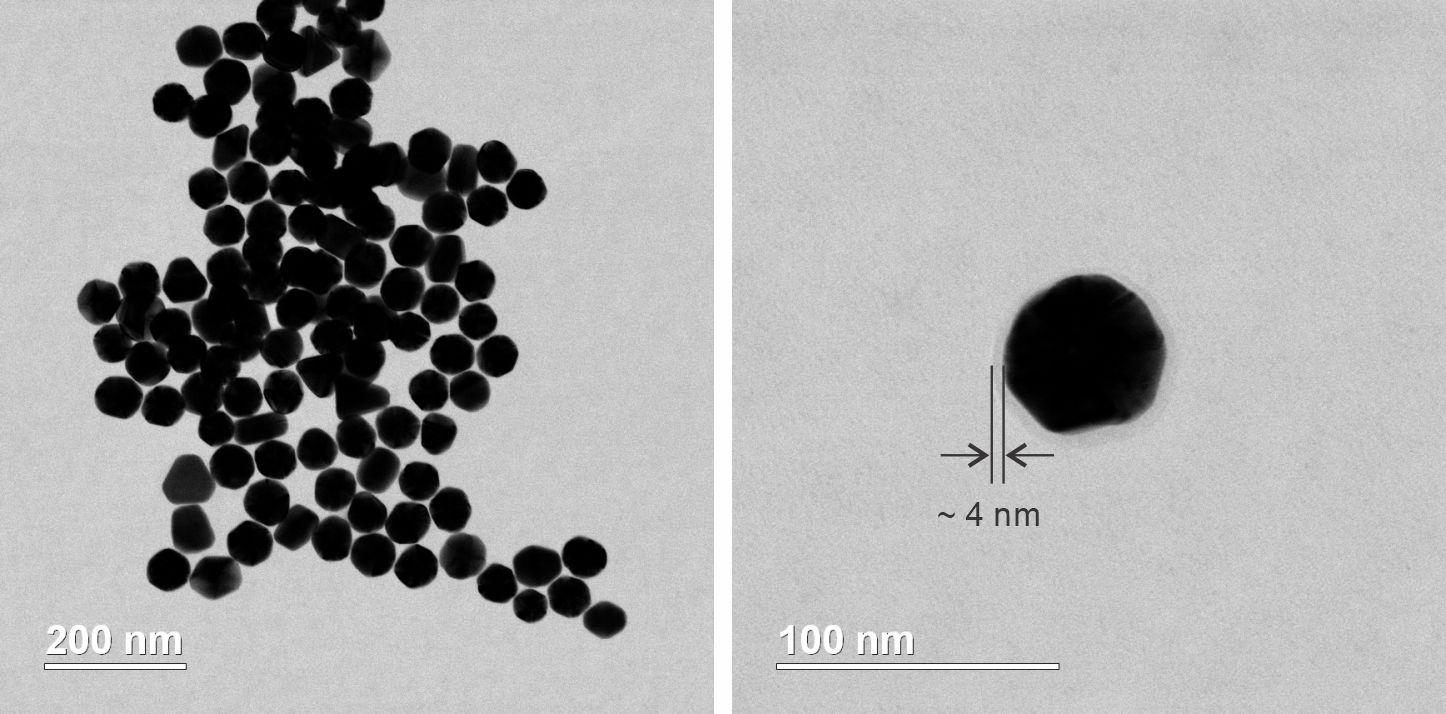


Figure S1. TEM images of 60 nm AuNPs used in this study. A uniform and thin organic ligand layer of approximately 4 nm can be observed in the high-resolution image (right).


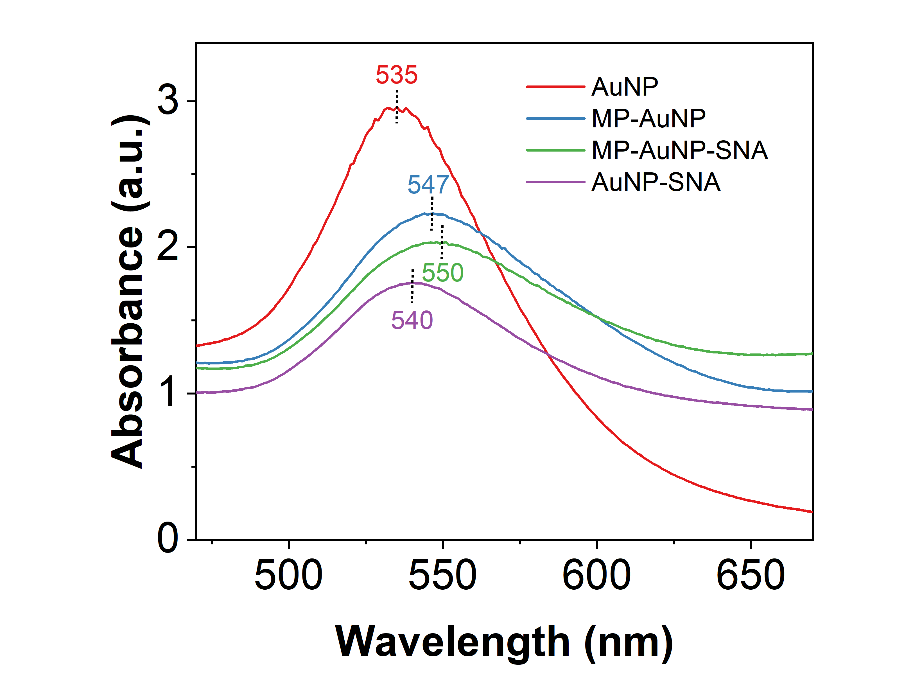


Figure S2. UV–vis spectra of AuNP, MP–AuNP, MP–AuNP–SNA, and AuNP–SNA. The observed red shifts in the peaks indicate successful stepwise surface modifications.


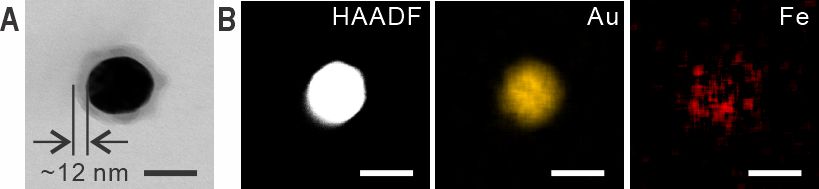


Figure S3. Characterization of MP–AuNP. (A) TEM image of an MP–AuNP, showing a visibly thicker and slightly rougher metal–phenolic coating compared to the organic ligand layer observed in Figure S1. (B) HAADF–STEM image and corresponding EDS elemental maps of an MP–AuNP. The colocalization of Au and Fe signals confirms successful surface modification with the metal–phenolic coating. Scale bars: 50 nm.


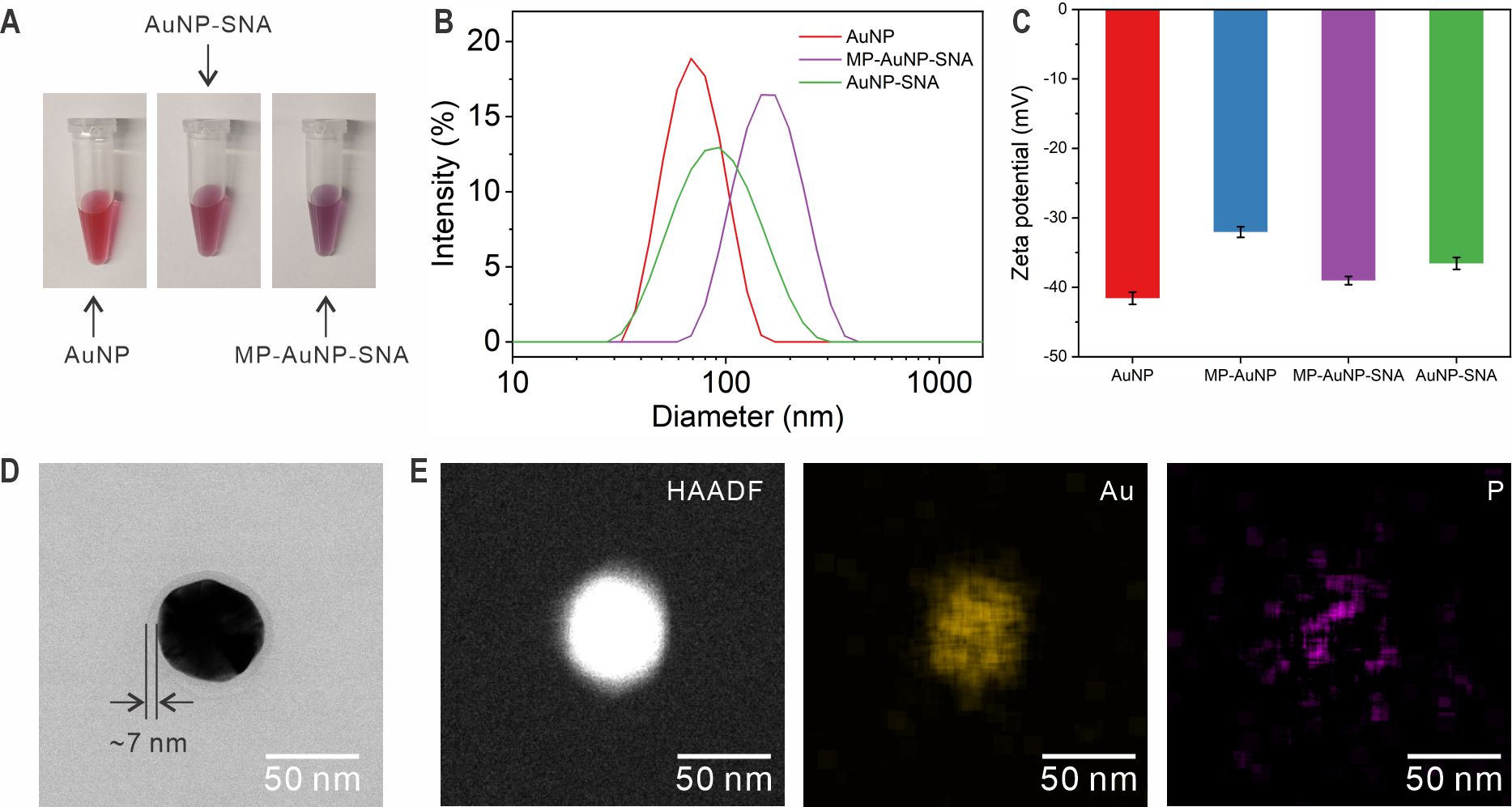


Figure S4. Characterization of AuNP–SNA. (A) Photographs of AuNP, AuNP–SNA, and MP–AuNP–SNA solutions. The MP–AuNP–SNA sample exhibits a more purple color than AuNP–SNA, consistent with the presence of the metal–phenolic coating. (B) DLS size distributions of AuNP, AuNP–SNA, and MP–AuNP–SNA. (C) Zeta potentials of AuNP, MP–AuNP, MP–AuNP–SNA, and AuNP–SNA. (D) TEM image of an AuNP–SNA. (E) HAADF–STEM image and corresponding EDS elemental maps of an AuNP–SNA, showing clear colocalization of Au and P signals, confirming successful surface modification with DNA strands.


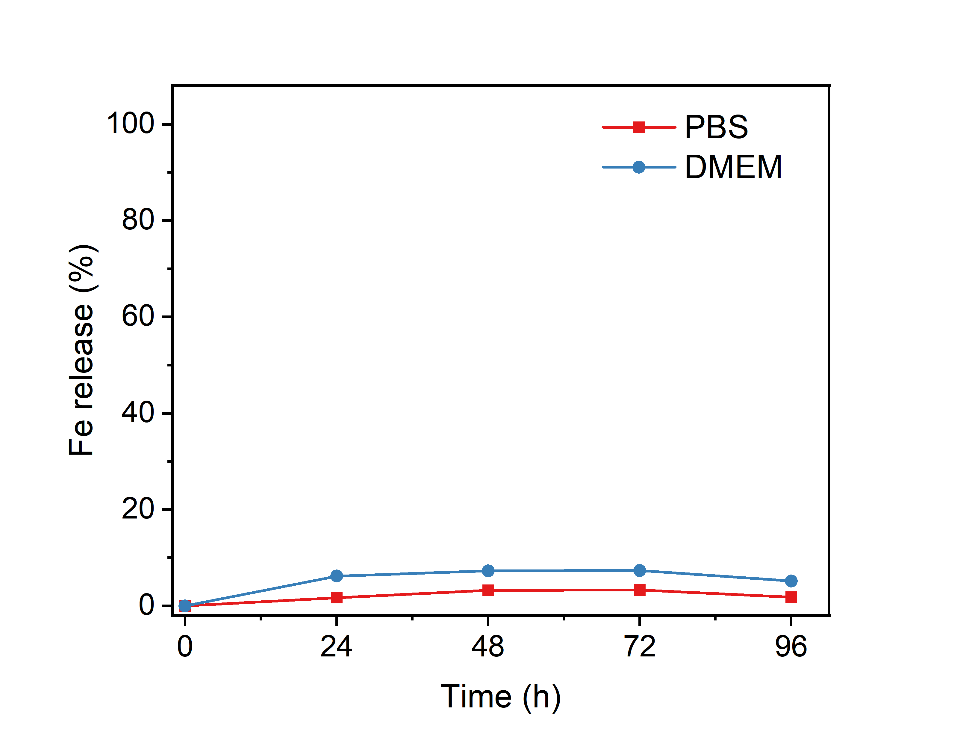


Figure S5. MP-AuNP-SNA exhibits minimal Fe release in 1× PBS and DMEM over 96 hours, confirming its robust stability under physiological and cell culture conditions.


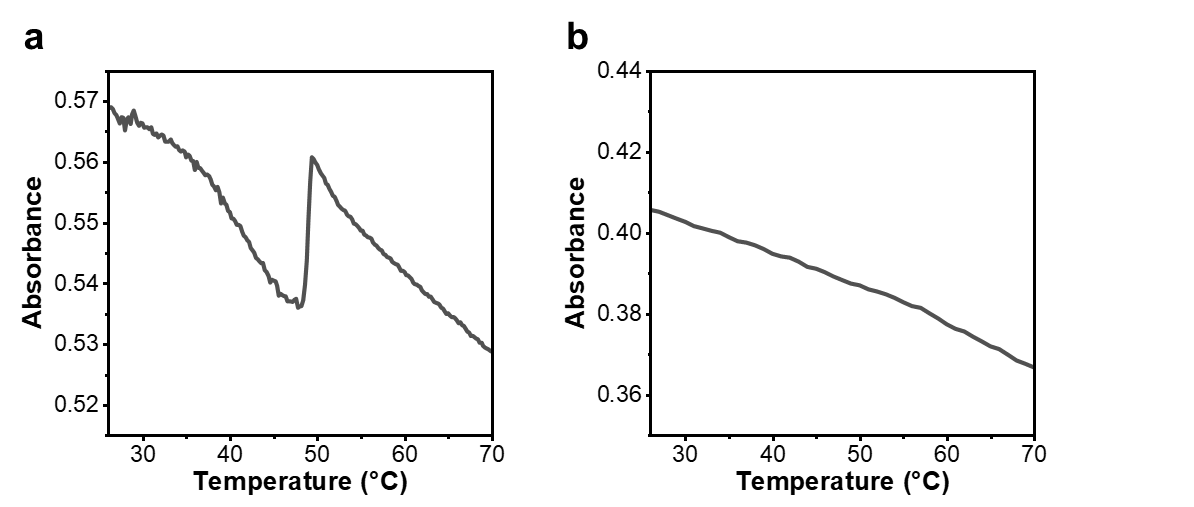


Figure S6. UV–vis thermal melting analysis of DNA-mediated MP-SNA assemblies with and without linker strands. In the presence of the complementary linker strands (a), the assembled MP-SNAs showed a temperature-dependent absorbance change with a clear transition region, whereas the control sample lacking linker strands did not exhibit a comparable melting transition and instead showed only a gradual monotonic decrease in absorbance (b).


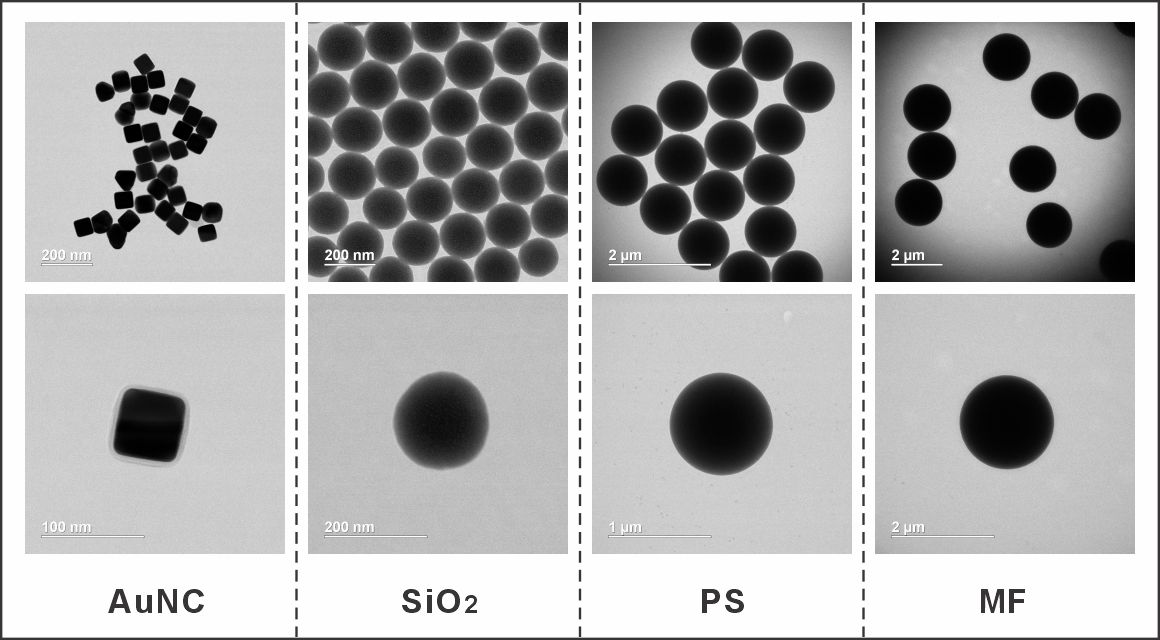


Figure S7. TEM images of unmodified AuNC, SiO_2_, PS, and MF particles, showing their uniform morphology and size distribution prior to surface modification.


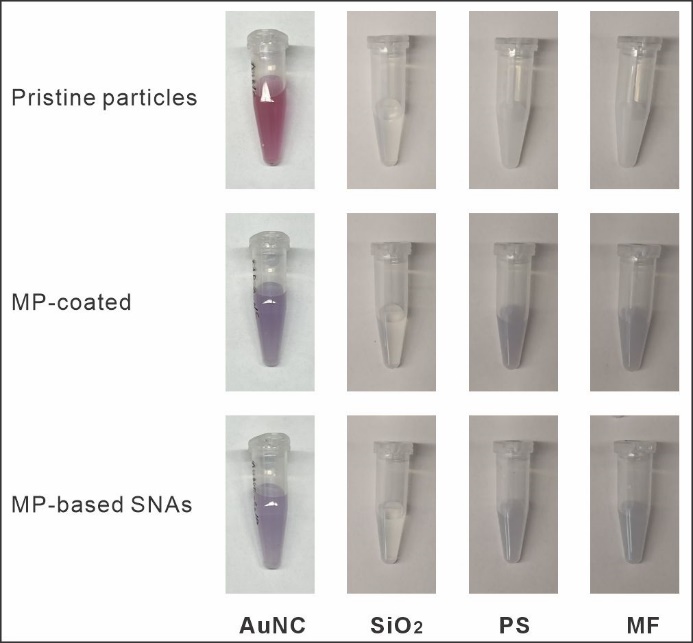


Figure S8. Photographs showing the appearance of different particles (AuNC, SiO_2_, PS, and MF) before and after MP coating and subsequent DNA conjugation. After MP coating, all particles exhibit a characteristic blue coloration, confirming the formation of the coordination networks.


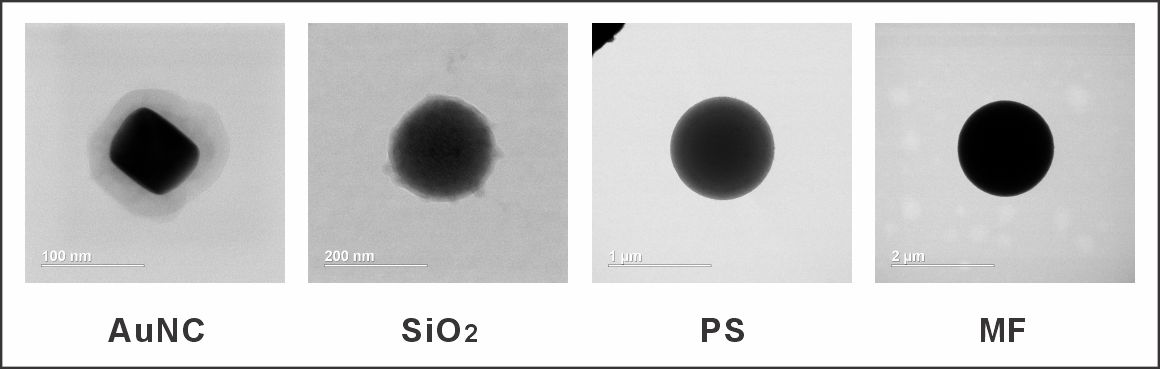


Figure S9. TEM images of various MP-coated particles (AuNC, SiO_2_, PS, and MF). The MP coating is clearly visible on the nanoscale particles (AuNC and SiO_2_), confirming successful surface modification.


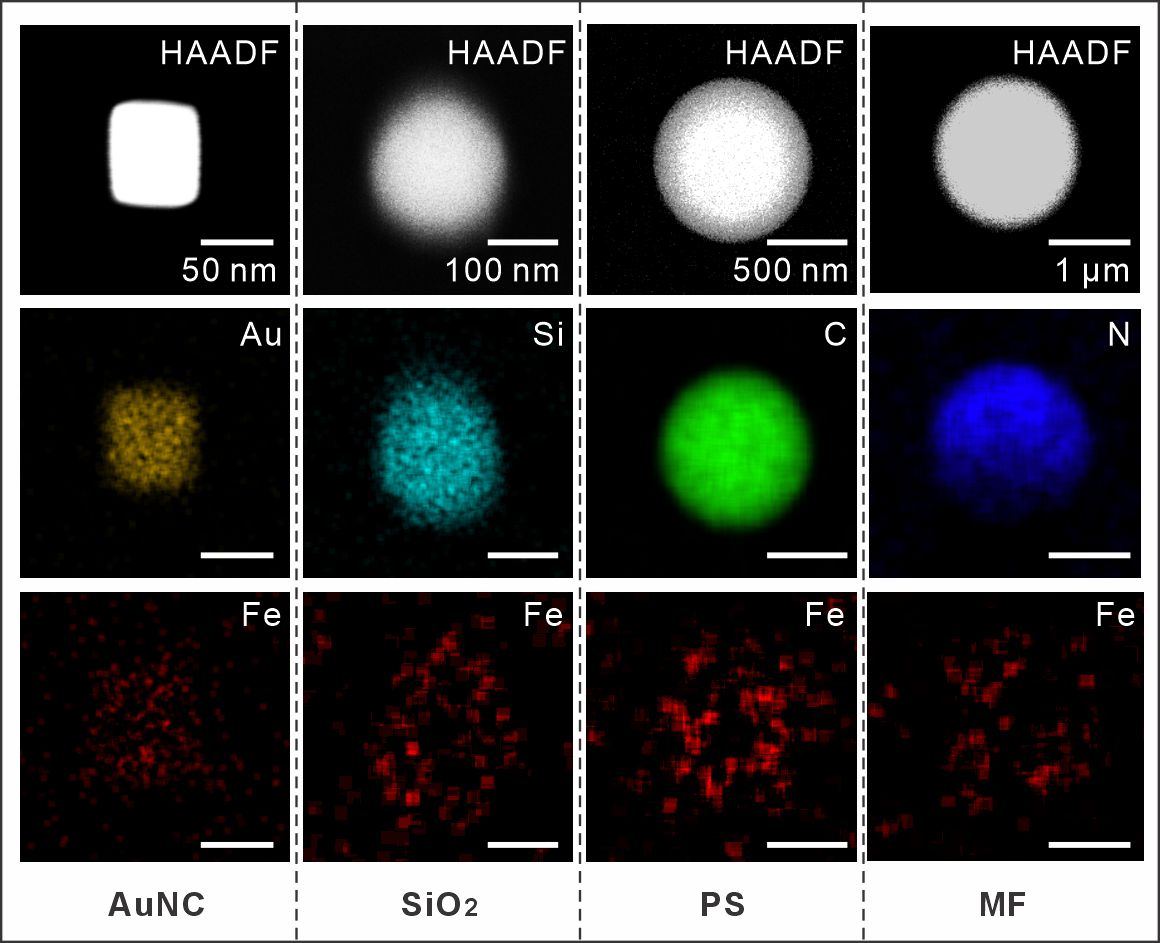


Figure S10. HAADF–STEM images and corresponding EDS elemental maps of various MP-coated particles (AuNC, SiO_2_, PS, and MF). The colocalization of Fe signals with the particles confirms successful surface modification with the metal–phenolic coating.


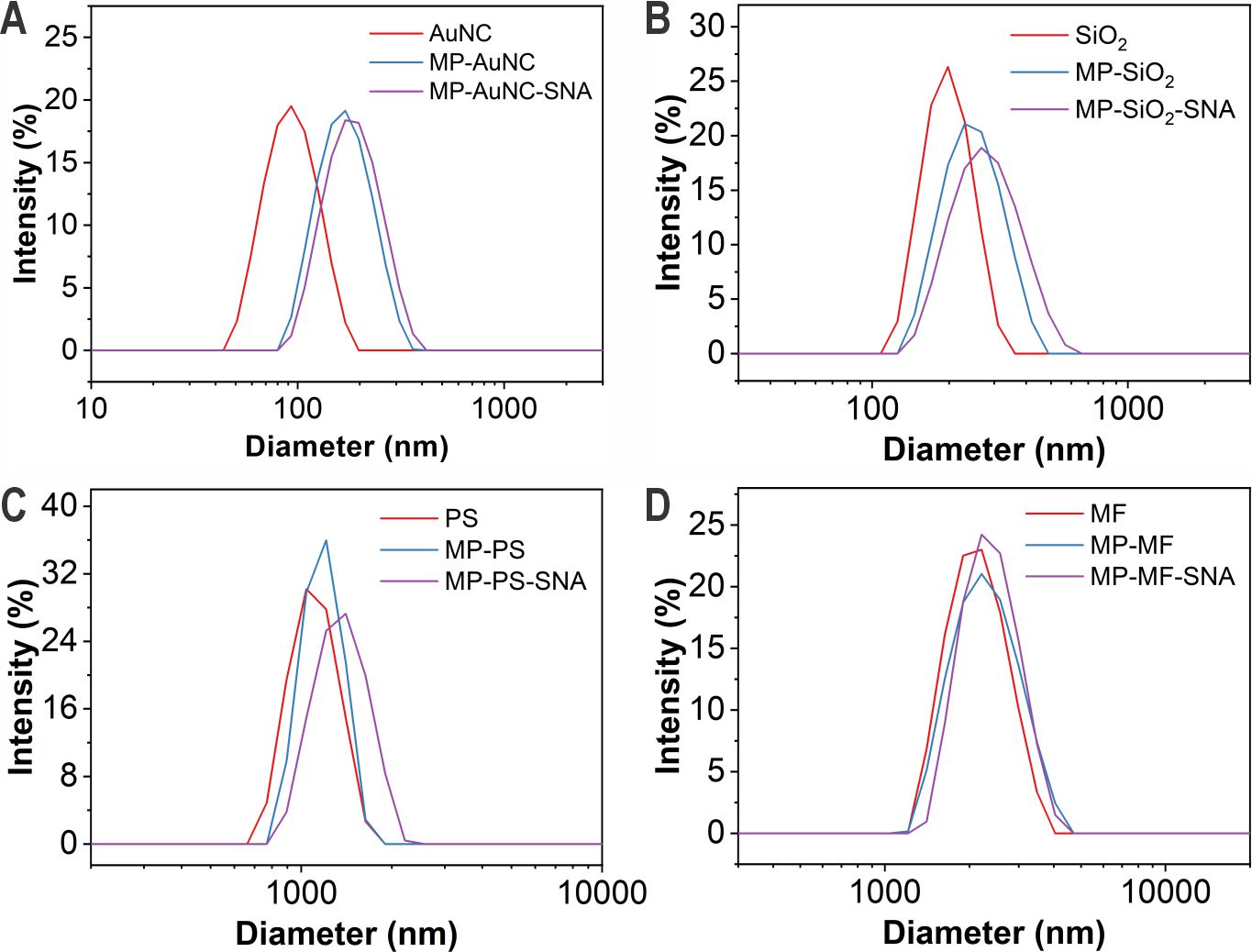


Figure S11. DLS size distributions of various particles before and after MP coating and subsequent DNA conjugation: (A) AuNC, (B) SiO_2_, (C) PS, and (D) MF. Quantitative size-distribution data based on DLS measurements reflect ensemble-average hydrodynamic size distributions in solution. In contrast, the TEM images in Figures S9 and S10 enable direct visualization of the core–shell structure and coating uniformity at the single-particle level. Together, DLS and TEM provide complementary information on particle size distribution and morphology, respectively.


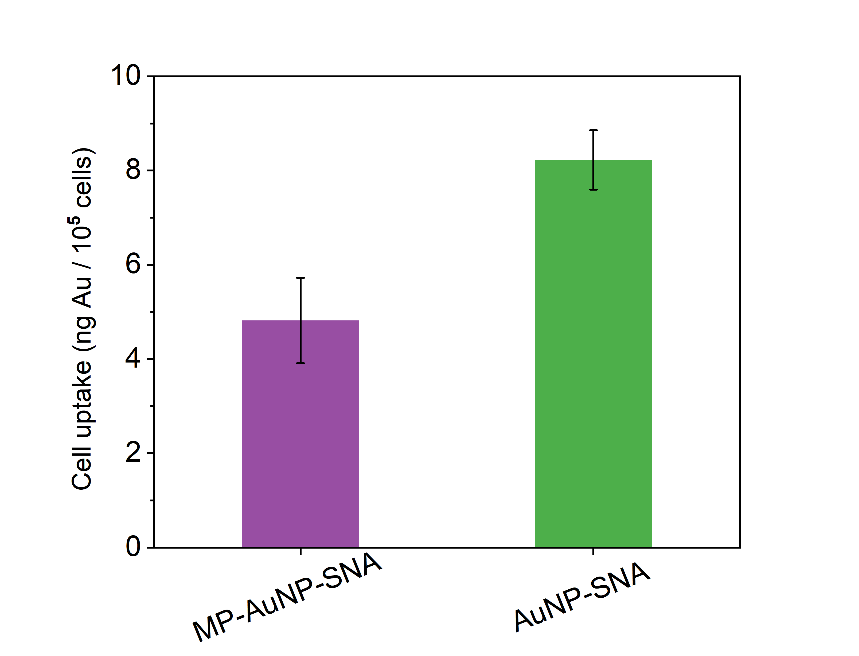


Figure S12. Comparison of cellular uptake of AuNP–SNA and MP–AuNP–SNA, as determined by ICP–MS analysis.


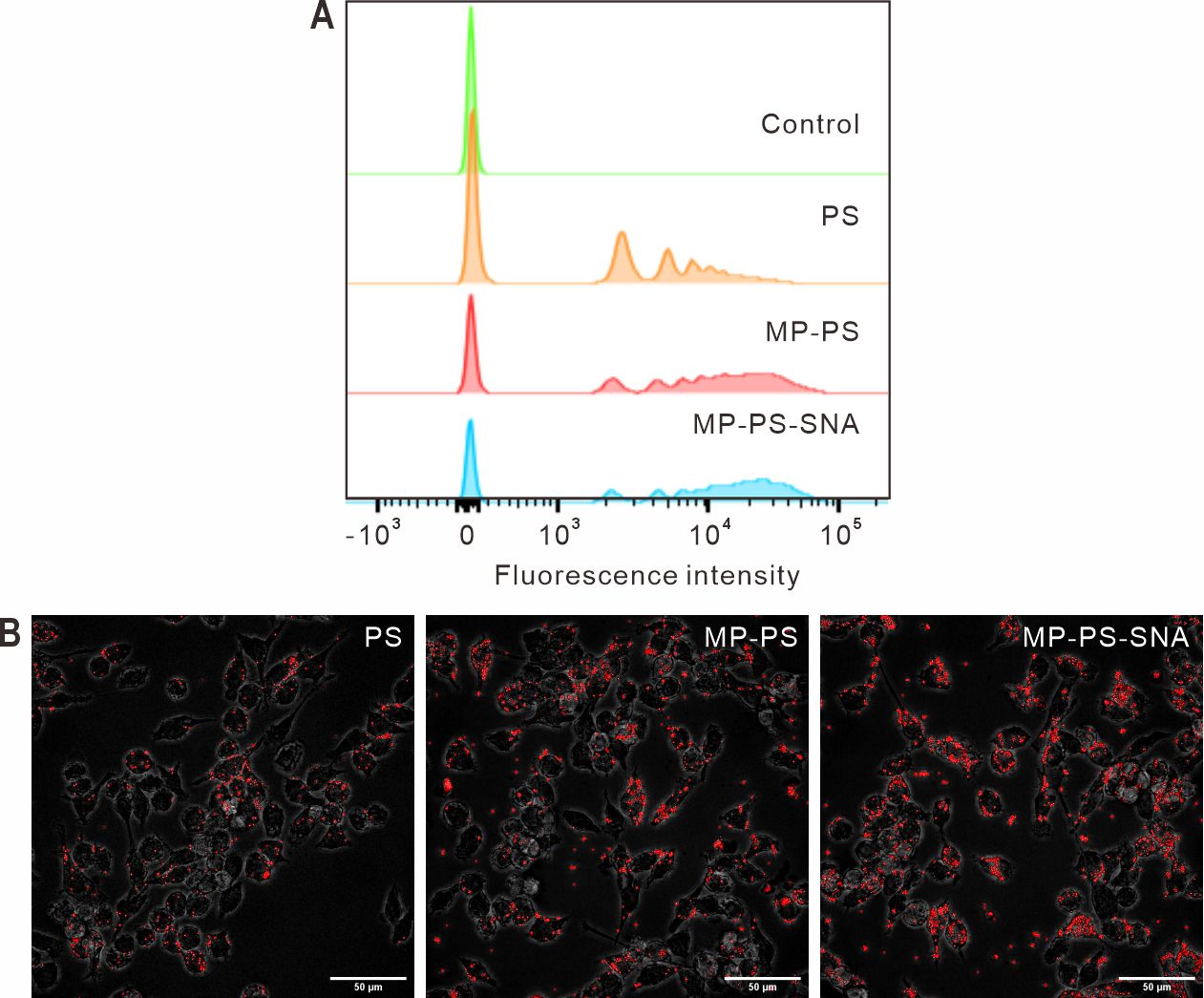


Figure S13. Comparison of cellular uptake of PS, MP–PS, and MP–PS–SNA particles. Uptake was assessed by (A) flow cytometry and (B) confocal fluorescence imaging, highlighting the effect of the DNA functionalization on particle internalization.


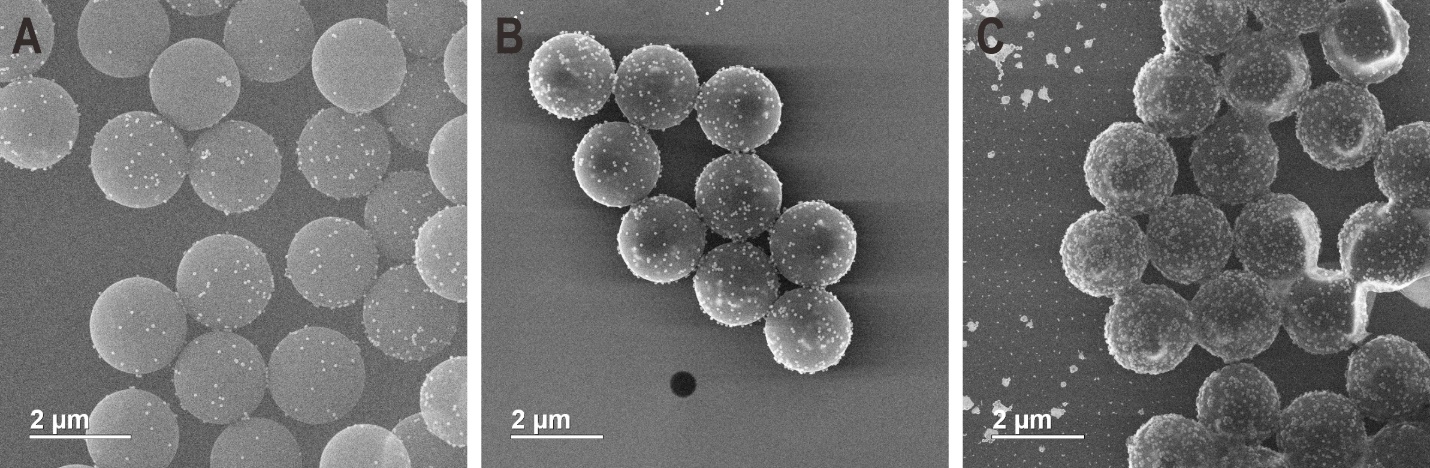


Figure S14. Large-area SEM images of core–satellite superstructures assembled from MF particles and AuNPs at different total surface area ratios: (A) 3:1, (B) 1:1, and (C) 1:4, illustrating the effect of particle ratio on assembly density and uniformity.


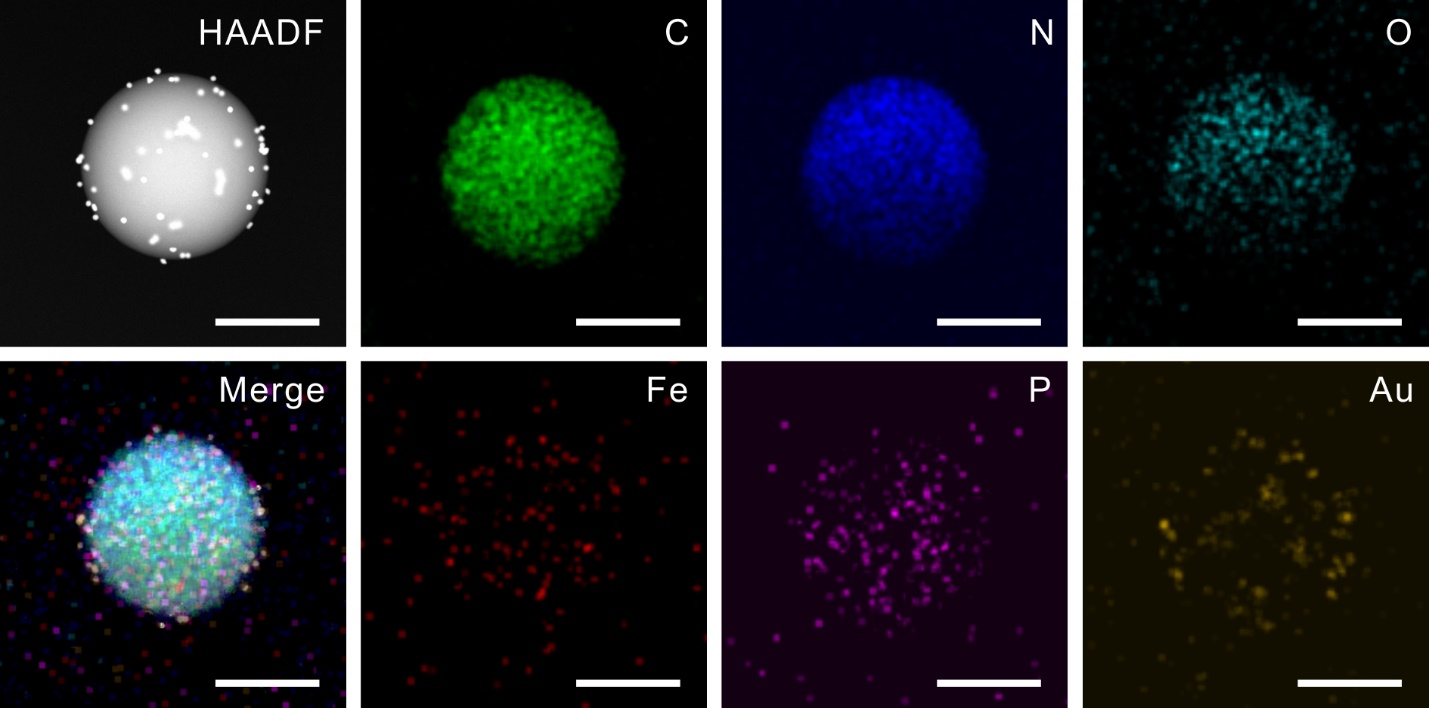


Figure S15. HAADF–STEM image and EDS elemental maps of a core–satellite superstructure assembled from MF particles and AuNPs at a 3:1 total surface area ratio, showing the number and spatial distribution of AuNPs on the surface of the MF particle.


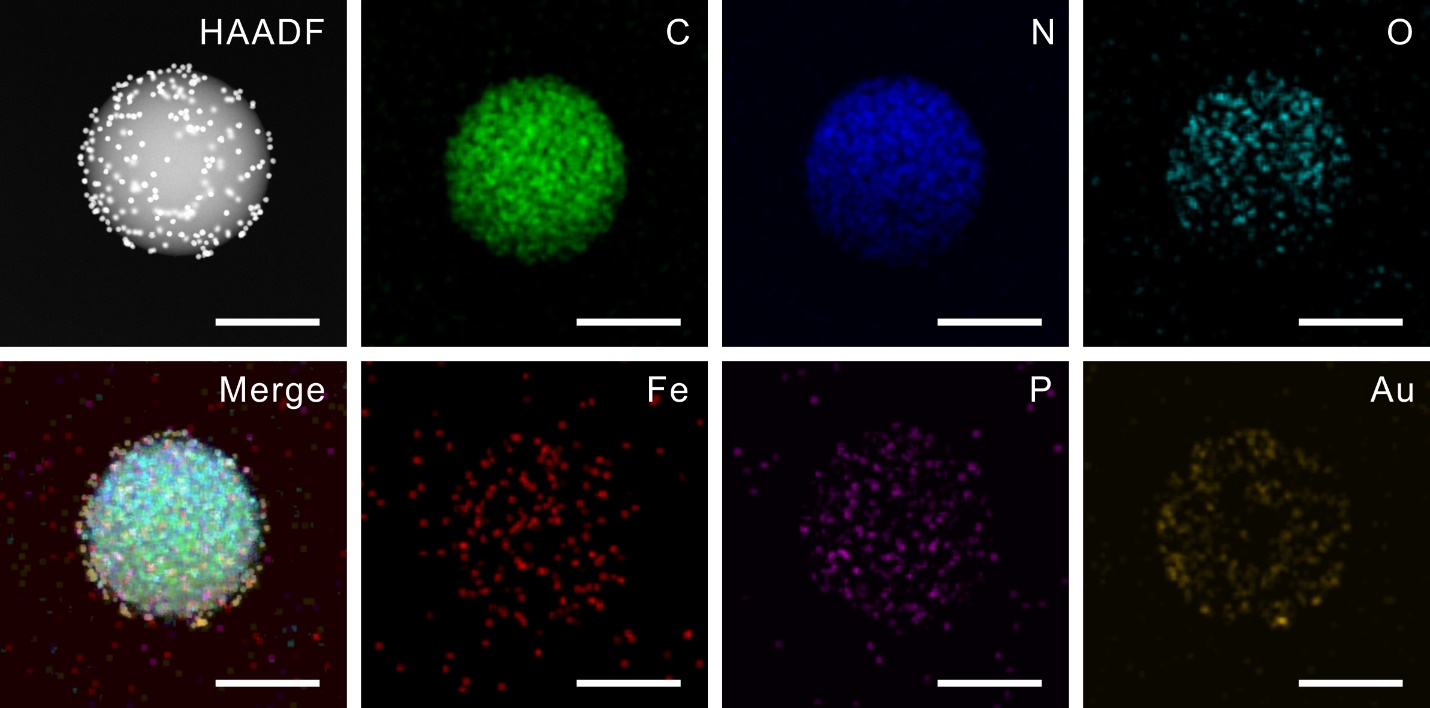


Figure S16. HAADF–STEM image and EDS elemental maps of a core–satellite superstructure assembled from MF particles and AuNPs at a 1:1 total surface area ratio, showing the number and spatial distribution of AuNPs on the surface of the MF particle.


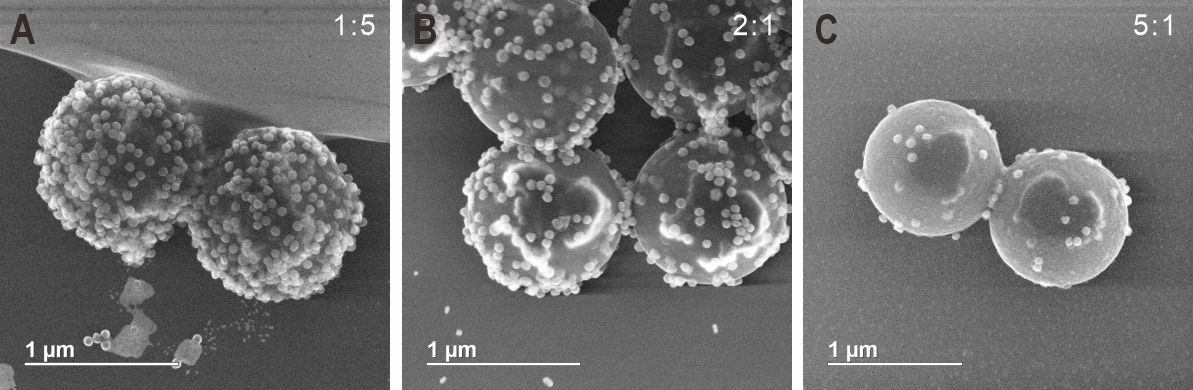


Figure S17. SEM images of core–satellite superstructures assembled from PS particles and AuNPs at different total surface area ratios: (A) 1:5, (B) 2:1, and (C) 5:1, illustrating the effect of particle ratio on assembly density and uniformity.


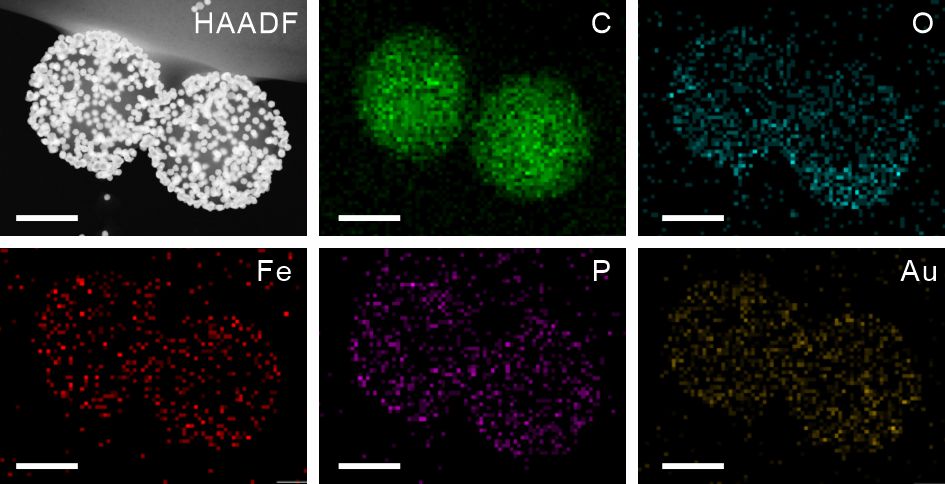


Figure S18. HAADF–STEM image and EDS elemental maps of core–satellite superstructures assembled from PS particles and AuNPs at a 1:5 total surface area ratio, showing the number and spatial distribution of AuNPs on the surface of the PS particles. Scale bars: 500 nm.


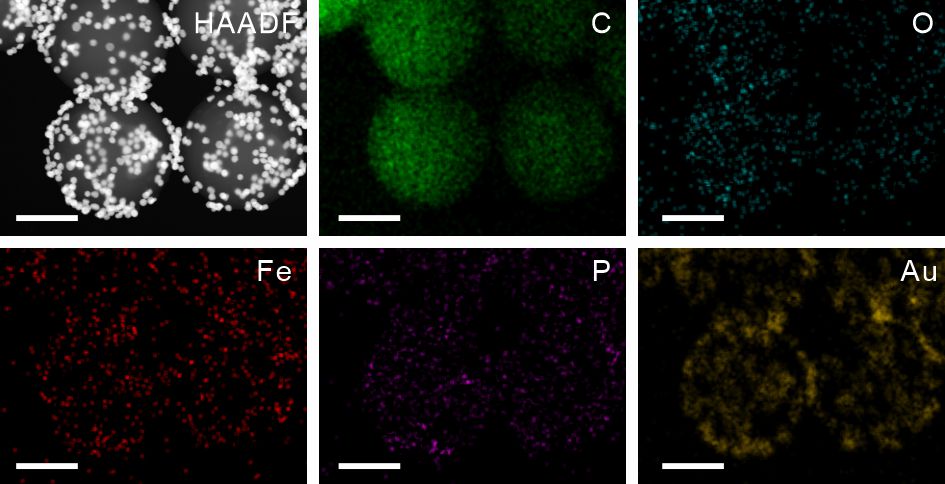


Figure S19. HAADF–STEM image and EDS elemental maps of core–satellite superstructures assembled from PS particles and AuNPs at a 2:1 total surface area ratio, showing the number and spatial distribution of AuNPs on the surface of the PS particles. Scale bars: 500 nm.


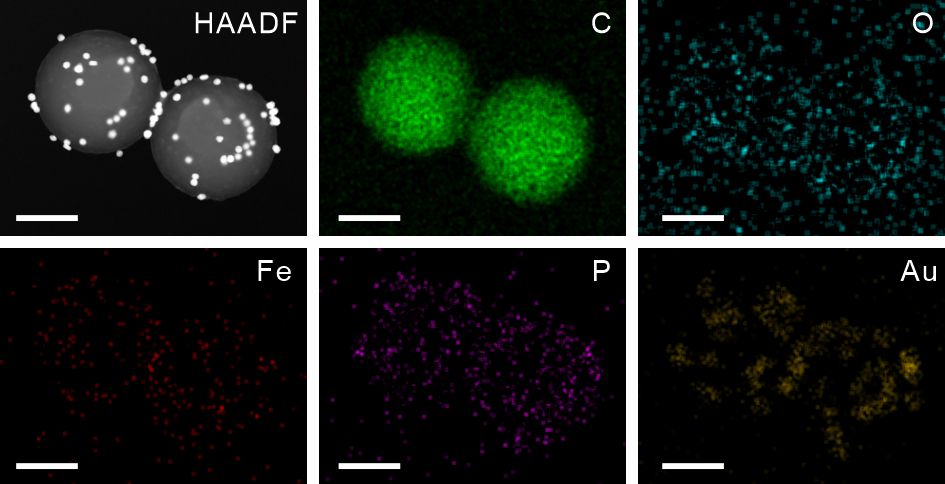


Figure S20. HAADF–STEM image and EDS elemental maps of core–satellite superstructures assembled from PS particles and AuNPs at a 5:1 total surface area ratio, showing the number and spatial distribution of AuNPs on the surface of the PS particles. Scale bars: 500 nm.

References

[1] M. N. O'Brien, M. R. Jones, K. A. Brown, C. A. Mirkin, “Universal Noble Metal Nanoparticle Seeds Realized Through Iterative Reductive Growth and Oxidative Dissolution Reactions,” *J. Am. Chem. Soc.* **2014**, *136*, 7603-7606.

[2] H. Ejima, J. J. Richardson, K. Liang, et al., “One-Step Assembly of Coordination Complexes for Versatile Film and Particle Engineering,” *Science* **2013**, *341*, 154-157.

[3] M. Björnmalm, J. W. Cui, N. Bertleff-Zieschang, et al., “Nanoengineering Particles through Template Assembly,” *Chem. Mater.* **2017**, *29*, 289-306.

[4] S. J. Hurst, A. K. R. Lytton-Jean, C. A. Mirkin, “Maximizing DNA loading on a range of gold nanoparticle sizes,” *Anal. Chem.* **2006**, *78*, 8313-8318.
